# Hodgkin’s lymphoma is a rare form of clonal haematological non-mast cell disease in systemic mastocytosis

**DOI:** 10.1186/s13000-015-0235-y

**Published:** 2015-03-14

**Authors:** Gorana Gasljevic, Biljana Grcar-Kuzmanov, Alenka Grosel, Matjaz Sever, Barbara Gazic, Veronika Kloboves-Prevodnik

**Affiliations:** Department of Pathology, Institute of Oncology, Zaloska 2, 1000 Ljubljana, Slovenia; Department of Laboratory medicine, Institute of Oncology, Zaloska 2, 1000 Ljubljana, Slovenia; Department of Hematology, University Medical Centre Ljubljana, 1000 Ljubljana, Slovenia; Department of Cytology, Institute of Oncology, Zaloska 2, 1000 Ljubljana, Slovenia

**Keywords:** Hodgkin’s lymphoma, Systemic mastocytosis, Systemic mastocytosis with associated clonal haematological non-mast cell lineage disease (SM-AHNMD)

## Abstract

**Background:**

The association of systemic mastocytosis (SM) with a non-mast cell haematological neoplasm represents a specific subtype of mastocytosis termed systemic mastocytosis with associated haematological non-mast cell disease (SM-AHNMD). The overwhelming majority of the associated neoplasms are of myeloid origin, while lymphoid neoplasms associated with SM have been reported rarely. Association of SM with Hodgkin’s lymphoma (HL) is exceedingly rare; so far, only two cases of HL as associated hematological non-mast cell disease in systemic mastocytosis have been published in the recent English literature.

**Case:**

We present a case of a 37-year-old otherwise healthy male who was referred to our institution because of a one-month lasting dysphagia of both hard and liquid food. Physical examination showed tumour in the left jugular area measuring 2 cm in the largest diameter while computer tomography of the thorax revealed a 5.2 cm large, hypodense, soft tissue tumour between the trachea and left arteria carotis communis. On the basis of FNAB findings, the diagnosis of a “neutrophil-rich” Hodgkin’s lymphoma was established. Excisional biopsy of mediastinal tumor showed lymphoid neoplasm with morphology and immunophenotype consistent with nodular sclerosis classical Hodgkin’s lymphoma (NScHL). Bone marrow trephine biopsy and the MGG-stained smear of the bone marrow aspirate performed for lymphoma staging revealed an existence of systemic mastocytosis which was unexpected and incidental finding. Mast cells were highlighted by CD117 and tryptase immunostainings while CD25 positivity of mast cells was consistent with their neoplastic phenotype.There were no HL infiltrates present in the bone marrow.

**Conclusion:**

We report a very rare combination of systemic mastocytosis with Hodgkin’s lymphoma as associated clonal haematological non-mast cell lineage disease. Systemic mastocytosis was an unexpected finding. The diagnosis of SM in bone marrow in our case was straight-forward, but it can be difficult in the case of reactive lymphoid aggregates or a difficult distinction between SM and HL infiltration. In particular, distinction can be challenging from the immunohistochemical point of view in the case of high-grade mast cell disease which can be CD30 positive.

## Background

Mastocytosis is the result of a clonal, neoplastic proliferation of mast cells that accumulate in one or more organ systems. It is characterized by the presence of multifocal clusters or aggregates of abnormal mast cells. In the last WHO classification [1] of tumours of haematopoietic and lymphoid tissues, seven subtypes of mastocytosis are defined by the distribution of the disease and its clinical manifestations: cutaneous mastocytosis (CM), indolent systemic mastocytosis (ISM), systemic mastocytosis with associated clonal haematological non-mast cell lineage disease (SM-AHNMD), aggressive systemic mastocytosis (ASM), mast cell leukaemia (MCL), mast cell sarcoma (MCS) and extracutaneous mastocytoma. In CM, mast cell infiltrates are restricted to the skin, whereas systemic mastocytosis (SM) is characterised by involvement of at least one extracutaneous organ with or without involvement of the skin. Bone marrow is almost always involved in SM [1].

The association of systemic mastocytosis with a non-mast cell haematological neoplasm represents a specific subtype of mastocytosis termed SM-AHNMD [1]. It is the second most frequent variant of SM [2]. The overwhelming majority of the associated neoplasms are of myeloid origin, while lymphoid neoplasms associated with SM have been reported rarely [3-6]. Association of SM with Hodgkin’s lymphoma has to be exceedingly rare; a review of the recent English literature on coexistence of Hodgkin’s lymphoma and systemic mastocytosis resulted in only two published articles so far [7,8]. The objective of this study is to report the clinical and pathological features of a 37-year-old male patient who was diagnosed with Hodgkin’s lymphoma of the upper mediastinum and indolent systemic mastocytosis which was found incidentally from bone marrow trephine biopsy performed for lymphoma staging.

## Case presentation

A 37-year-old otherwise healthy male was referred to our institution because of a one-month lasting dysphagia of both hard and liquid food. He denied any breathing difficulties. There was no evidence of systemic symptoms. Physical examination showed tumour in the left jugular area measuring 2 cm in the largest diameter and some small, unsuspicious lymph nodes on both sides of the neck. The liver and spleen were not enlarged. There were no skin lesions. Peripheral blood counts were within normal limits. Serologies for anti-HAV, HbsAg, anti- HBc, anti-Hbs, anti-HCV and HIV were all negative. Lately performed laboratory studies revealed that the patient’s serum tryptase level was 79 ng/ml (normal 2–10). Fine-needle aspiration biopsy (FNAB) of jugulare tumour was performed as part of the initial diagnostic procedure, but the sample obtained was not diagnostic. Computer tomography of the thorax revealed a 5.2 cm large, hypodense, soft tissue tumour between the trachea and left arteria carotis communis, and subsequent ultrasound-guided FNAB was performed. The tumour was situated at the level of the upper thoracic opening. Cytologically, the diagnosis of a “neutrophil-rich” Hodgkin’s lymphoma was established. Excisional biopsy of the tumour was performed to confirm the cytological findings. Bone marrow involvement with lymphoma was assessed by aspiration and trephine biopsy.

### Cytological and pathological findings

**Figure 1:**
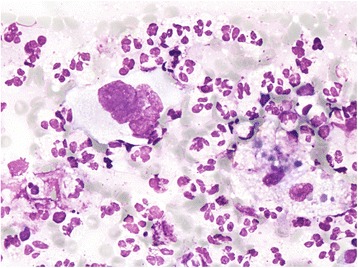
**Reed-Sternberg cells were rare and mainly found at the edge of the smear (Giemsa, 60X).**

The Giemsa-stained smear of the mediastinal tumour was very cellular. Neutrophils predominated, obscuring a few tumour cells found mainly along the edge of the smear. Tumour cells which were not degenerated showed moderately abundant, pale cytoplasm and single or multiple lobulated nuclei (Figure 1). Chromatin was finely granular. Nucleoli were conspicuous only in some cells. In a dense neutrophilic background, some histiocytes, few eosinophils, fibrin fibres and proteinaceous granular material were also observed. Because of the typical cytological picture and despite the fact that additional immunophenotypic analyses could not be performed (due to lack of tumour cells in the slides prepared for immunocytochemistry), the diagnosis of a “neutrophil-rich” Hodgkin’s lymphoma was suggested.

**Figure 2:**
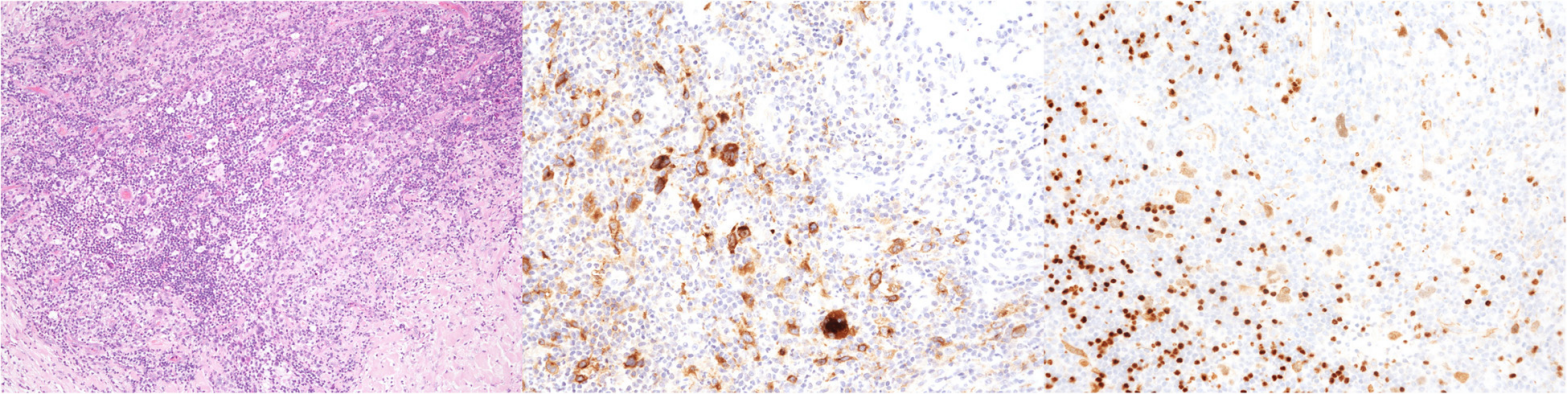
**H&E staining of mediastinal tumor, 10X; CD 30 and PAX 5 positive HRS cells, 20X.**

Excisional biopsy of the mediastinal tumour revealed a lymphoid neoplasm composed of relatively rare mononuclear Hodgkin’s and binuclear Reed-Sternberg cells residing in an infiltrate consisting of neutrophils, small lymphocytes, plasma cells, eosinophils and histiocytes (Figure 2). Some of the H-RS cells showed a retraction of the cytoplasmic membrane, creating the appearance of the so-called lacunar cells. Focally, HRS cells had more lobulated nuclei with smaller lobes. On the periphery of the specimen, a broad band of fibrosis was present. Large atypical cells were immunohistochemically positive for CD30, CD15, Pax5 and MUM1 (Figure 2). In situ hybridisation for EBV was negative. Based on the morphology of the H-RS cells, reactive infiltrate and present fibrosis, the tumour was classified as nodular sclerosis classical Hodgkin’s lymphoma (NScHL).

**Figure 3:**
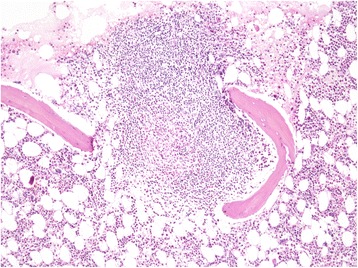
**Trephine bone marrow biopsy – mastocyte infiltrate, H&E 40X.**

**Figure 4:**
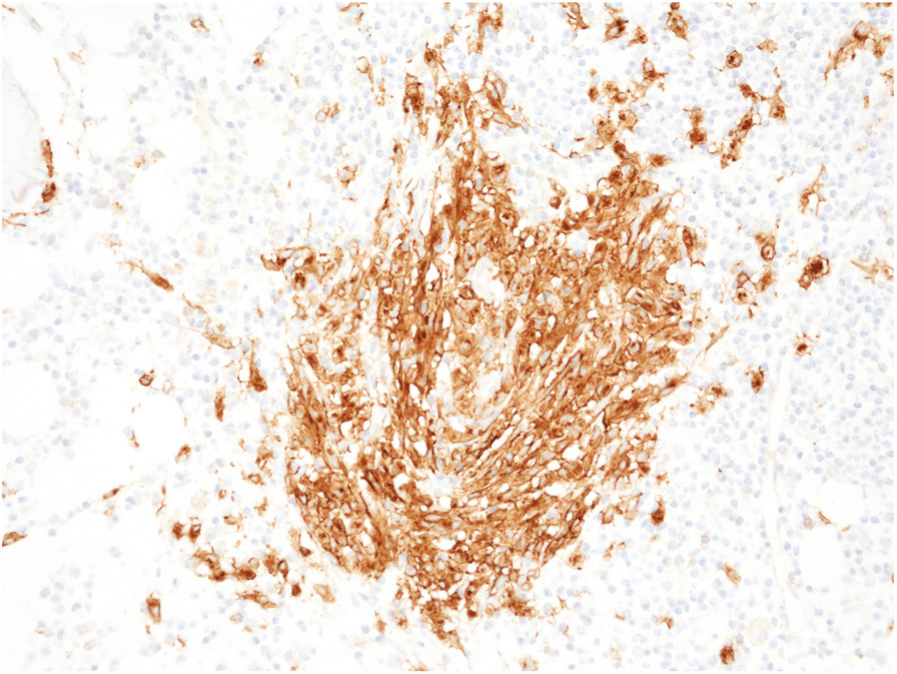
**Mastocytes highlighted by immunostaining for CD 117, 20X.**

**Figure 5:**
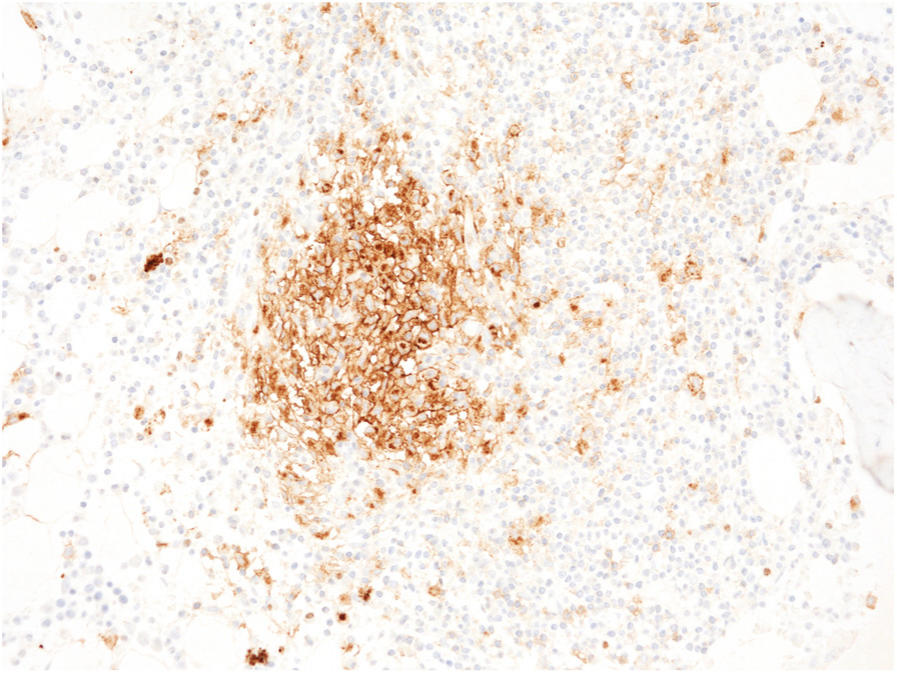
**Abberant expression of CD25 on mastocyte cells, 60X.**

Bone marrow trephine biopsy and the MGG-stained smear of the bone marrow aspirate performed for lymphoma staging revealed a slightly hypercellular bone marrow with some smaller clusters of atypical mast cells and a large peritrabecular infiltrate composed of atypical mastocytes and lymphocytes (Figure 3). Mast cells were highlighted by CD117 and tryptase immunostainings (Figure 4). CD25 and CD2 positivity of mast cells was consistent with their neoplastic phenotype (Figure 5). There were no HL infiltrates present in the bone marrow.

## Discussion

According to the current WHO classification, patients with SM and concurrent haematological malignancy, either myeloid or lymphoid, are included in the group of so-called SM-AHNMD. Neoplasms associated with SM are mainly of myeloid origin, while lymphoproliferative diseases (LPD) are described only occasionally. It has been shown that in the case of SM associated with myeloid malignancies, the neoplastic mastocytes and malignant myeloid cells are clonally related, and both diseases are often diagnosed concurrently [9,10]. In contrast, patients with SM and LPD usually have a long history of indolent SM; LPD and SM clones are distinct [4]. The present case of SM-cHL is interesting in several aspects. First of all, the cytological picture of the mediastinal tumour was unusual because it mimicked suppurative lymphadenitis. Secondly, coexistence of HL and SM is exceedingly rare. To the best of the authors’ knowledge, only two cases have been described in the English literature so far. Last but not least, patients with SM and LPD usually have a long history of indolent SM, which was not present in our patient.

From the cytological aspect, only a few cases of Hodgkin’s lymphoma with numerous neutrophils have been described in the literature so far [11-14]. Histologically, these cases have been mostly classified as nodular sclerosis cHL, and only few of them as mixed cellularity cHL [11-14]. Such cases could be challenging because a predominance of neutrophils and presence of necrosis can obscure the diagnostic Reed-Sternberg and Hodgkin cells, and they can be misdiagnosed as suppurative lymphadenitis [11-14]. To avoid a false-negative cytological diagnosis, an accurate search for Reed-Sternberg and Hodgkin cells must be carry out. In the differential diagnosis of “neutrophil-rich” HL and suppurative lymphadenitis, “neutrophil-rich” anaplastic large T-cell lymphoma (ALCL) and metastatic carcinoma must be considered. Suppurative lymphadenitis is most commonly found in tuberculosis, Calmette Guerin bacillus and atypical mycobacterial infection, Kikuchi’s disease, cat-scratch disease, and lymphogranuloma venereum [13,14]. Identification of the infectious agent is crucial for the diagnosis, therefore a part of the FNAB sample must be sent for microbiological testing. “Neutrophil-rich” ALCL may be difficult to differentiate from “neutrophil-rich” Hodgkin’s lymphoma. Immunocytochemical staining for CD30, CD15, ALK, CD45, CD20, EMA and T-cell markers can resolve this differential diagnostic dilemma [15,16]. Metastatic carcinoma could also represent a differential diagnostic possibility. Metastatic squamous-cell carcinoma is particularly prone to necrosis and inflammation, which in many cases leads to the cytological picture that can be very similar to that in “neutrophil-rich” HL or ALCL [14]. However, this dilemma could be easily solved by cytokeratin immunocytochemical staining.

Coexistence of SM with HL might be challenging also in terms of separation of bone marrow infiltrates. Reactive lymphoid aggregates are a feature of bone marrow infiltrates in patients with indolent SM. They can be composed of a mixture of mast cells and lymphocytes, or either of them may predominate [17]. As well, increased eosinophils can accompany lymphocytes as a part of the background infiltrate not only in systemic mastocytosis but also in HL. Since HL is also characterised by a paucity of neoplastic cells in comparison to background reactive cells, morphological similarity between the infiltrates of SM and HL could cause confusion; either of them can be misdiagnosed. Furthermore, aggressive mastocytosis (ASM) is morphologically characterised by a marked degree of bone marrow infiltration that can be diffuse or focal. Its major differential diagnosis is SM-AHNMD since AHNMD may be almost obscured by the massive mast cell infiltrate [18]. Besides, it has been shown that a major proportion of cases with high-grade SM (ASM, SM-AHNMD and mast cell leukaemia) show a strong expression of CD30 by a majority of neoplastic mast cells [18]. All of the above-mentioned, as well as CD30 positivity of mast cells, may result in the misinterpretation of bone marrow infiltration by HL, unless antitryptase staining is performed, and may contribute to erroneous tumour staging and treatment.

## Conclusions

In conclusion, we report a very rare combination of systemic mastocytosis with Hodgkin’s lymphoma as associated clonal haematological non-mast cell lineage disease. In our case, HL was presented as a mediastinal tumour and sub-classified as nodular sclerosis. Systemic mastocytosis was an unexpected finding. To our knowledge, this is the third reported case in the literature of such disease combination.

## Consent

Written informed consent was obtained from the patient for publication of this Case Report and any accompanying images. A copy of the written consent is available for review by the Editor-in-Chief of this journal.

## Abbreviations

CM: Cutaneous mastocytosis
ISM: Indolent systemic mastocytosis
SM-AHNMD: Systemic mastocytosis with associated clonal haematological non-mast cell lineage disease
ASM: Aggressive systemic mastocytosis
MCL: Mast cell leukaemia
MCS: Mast cell sarcoma
FNAB: Fine-needle aspiration biopsy
NScHL: Nodular sclerosis classical Hodgkin’s lymphoma
MGG: May Grinwald Giemsa
LPD: Lymphoproliferative diseases
ALCL: Anaplastic large T-cell lymphoma
cHL: Classical Hodgkin’s lymphoma
